# Antibacterial and Antifungal Activity of Essential Oils against Pathogens Responsible for Otitis Externa in Dogs and Cats

**DOI:** 10.3390/medicines4020021

**Published:** 2017-04-21

**Authors:** Valentina V. Ebani, Simona Nardoni, Fabrizio Bertelloni, Basma Najar, Luisa Pistelli, Francesca Mancianti

**Affiliations:** 1 Department of Veterinary Science, University of Pisa, viale delle Piagge 2, 56124 Pisa, Italy; simona.nardoni@unipi.it (S.N.); fabrizio.bertelloni@vet.unipi.it (F.B.); francesca.mancianti@unipi.it (F.M.); 2 Centro Interdipartimentale di Ricerca “Nutraceutica e Alimentazione per la Salute”, University of Pisa, via del Borghetto 80, 56124 Pisa, Italy; luisa.pistelli@unipi.it; 3 Department of Pharmacy, University of Pisa, via Bonanno 6, 56126 Pisa, Italy; basmanajar@hotmail.fr

**Keywords:** otitis externa, essential oils, antibacterial activity, antifungal activity, dogs, cats

## Abstract

**Background:** Essential oils (EOs) are recommended by some veterinarians to treat otitis externa in pets, but data about their efficacy in scientific literature are very scant. **Methods:** Nine commercial EOs, from roman chamomile (*Anthemis nobilis* L.), star anise (*Illicium verum*), lavender (*Lavandula hybrida*), litsea (*Litsea cubeba* (Lour.) Pers.), basil (*Ocimum basilicum* L.), oregano (*Origanum vulgare* L. subsp. *hirticum*), rosemary (*Rosmarinus officinalis* L.), clary sage (*Salvia sclarea* L.), and thyme (*Thymus vulgaris* L.) were tested against bacterial and fungal pathogens previously isolated from dogs and cats with otitis externa. In particular, the analyses were carried out against *Pseudomonas aeruginosa, Staphylococcus aureus, Staphylococcus pseudointermedius*, *Aspergillus niger, Aspergillus fumigatus, Aspergillus terreus, Candida albicans, Candida tropicalis, Trichosporon* sp., and *Rhodotorula* sp. **Results:** *O. vulgare* and *S. sclarea* showed superior antibacterial activity, even if not against all the strains. *Trichosporon* sp., *C. albicans*, and *A. terreus* were insensitive to most Eos, while other yeasts and molds showed different degrees of sensitivity. In particular, most fungi were inhibited by *O. vulgare* and *R. officinalis*. **Conclusions:** The obtained results suggest that some EOs could be included in treatment as an alternative therapeutic option in bacterial otitis complicated by fungi, in association with conventional drugs.

## 1. Introduction

Otitis externa is an inflammation of the external ear canal. Several predisposing factors in dogs and cats would be anatomic breed conformation, immunologic and endocrinopathic conditions, foreign material, trauma, and treatments. Various microorganisms can colonize the external ear canal, and they can proliferate with damage or inflammation due to primary factors [1]. Bacteria, yeasts, and parasites are common causes of otitis externa.

Among bacteria, staphylococci—mainly *Staphylococcus pseudointermedius* (formerly *Staphylococcus intermedius* [2]) and *Staphylococcus aureusx*—and *Pseudomonas* spp. are often isolated from ears of dogs and cats with acute or chronic otitis externa. 

About 27% of cases of otitis externa are reported as being caused by fungi, mainly by *Malassezia pachydermatis* [3]. Other fungi more or less frequently involved are *Aspergillus niger, Aspergillus fumigatus, Aspergillus terreus* [4], and several yeasts, such as *Candida albicans* [5,6], *Candida parapsilosis, Trichosporon cutaneum*, and *Cryptococcus laurentii* [7], as well as *Candida tropicalis* [6,8] and *Rhodotorula* [9].

Antibiotic and/or antifungal treatments do not always resolve otitis externa in pets; conversely, they can worsen the situation, causing secondary and recurring infections.

The use of some essential oils (EOs) against otitis externa in pets is currently recommended by some veterinarians that—based on their experience—obtained good results in the treatment of these infections. However, data about the efficacy of EOs against bacteria and yeasts related to canine and/or feline otitis are not available in the scientific literature, with the exception of reports on *M. pachydermatis* otitis externa [10]; furthermore, information about human cases are very scant [11,12,13].

The aim of this study was to evaluate the in vitro antibacterial and antifungal activities of EOs against the most frequent pathogens isolated from the ears of dogs and cats with otitis externa. EOs have been selected for their effectiveness as reported in literature and for their availability on the market. 

## 2. Material and Methods

### 2.1. Essential Oils

The study was performed employing the following nine EOs: roman chamomile (*Anthemis nobilis* L.), star anise (*Illicium verum*), lavender (*Lavandula hybrida*), litsea (*Litsea cubeba* (Lour.) Pers.), basil (*Ocimum basilicum* L.), oregano (*Origanum vulgare* L. subsp. *hirticum*), rosemary (*Rosmarinus officinalis* L.), clary sage (*Salvia sclarea* L.), and thyme (*Thymus vulgaris* L.). 

All EOs were acquired from the producer (FLORA^®^, Pisa, Italy). They were maintained at 4 °C in dark glass vials and were microbiologically analyzed for quality control before use. 

### 2.2. Gas Chromatography–Mass Spectrometry Analysis

The GC analysis was performed as previously described [14]. 

### 2.3. Antibacterial Activity

#### 2.3.1. Bacterial Strains

Each EO was tested against three bacterial strains: *Pseudomonas aeruginosa, S. aureus*, and *S. pseudointermedius*. All the isolates were previously obtained from cases of otitis externa of dogs and/or cats, typed, and stored at −80 °C in glycerol broth.

#### 2.3.2. Agar Disc Diffusion Method

Antibacterial activity of each EO was tested by Kirby-Bauer agar disc diffusion method following the previously described procedures [15,16].

Bacterial strains were also tested by Kirby-Bauer method to evaluate their in vitro sensitivity to the following antibiotics (Oxoid): amikacin (30 µg), amoxycillin–clavulanic acid (30 μg), ampicillin (10 µg), cefotaxime (30 µg), ceftazidime (30 µg), cephalexin (30 µg), ciprofloxacin (5 µg), erythromycin (10 µg), enrofloxacin (5 µg), streptomycin (10 µg), sulphametoxazole–trimethoprim (25 µg), and tetracycline (30 µg). The results were interpreted as indicated by the National Committee for Clinical Laboratory Standards (NCCLS) [17]. 

#### 2.3.3. Minimum Inhibitory Concentration 

Each bacterial strain resulting sensitive to the EOs in the Kirby-Bauer test was submitted to the determination of the minimum inhibitory concentration (MIC).

MIC was tested with agar disc diffusion method, adding 10 µL of 10%, 5%, 2.5%, 1.25%, 0.62%, 0.31% (*v*/*v*) of each EO. Results were successively calculated as µg/µL. All tests were performed in triplicate.

### 2.4. Antimycotic Activity

#### 2.4.1. Fungal Strains

Clinical isolates of *A. niger, A. fumigatus, A. terreus*, *C. albicans*, *C. tropicalis, Trichosporon* sp., and *Rhodotorula* sp. obtained from dogs and cats suffering from otitis externa were employed for testing. Molds were stored at −20 °C on potato dextrose agar, and yeasts were maintained in distilled water at room temperature.

#### 2.4.2. Microdilution Test

MICs were determined by a microdilution test carried out as reported elsewhere [15], starting from a dilution of 5% (*v*/*v*), following the methods described by Clinical and Laboratory Standards Institute M38A_2_ [18] for molds and Clinical and Laboratory Standards Institute M27A_3_ [19] for yeasts.

## 3. Results

### 3.1. Gas Chromatography–Mass Spectrometry Analysis

The composition of the assayed EOs is presented in Table 1. All these oils were rich in monoterpenes, except for *A. nobilis*, where non-terpenic esters such as isobutyl angelate (34.5%) and isoamyl angelate (18.7%) were the most represented constituents. The main terpenes identified in the EO of *O. vulgare* and *T. vulgaris* were carvacrol (65.9%) and thymol (52.6%), respectively, followed by p-cymene (15.3%) only in *T. vulgaris*. Linalyl acetate was evidenced in *S. sclarea* as the main constituent (54.7%). Linalool was the most important compound in the EO of *O. basilicum* and *L. hybrida* (46.0% and 31.5%, respectively). It is also important to highlight the presence of eugenol in *O. basilicum* with a significant percentage (11.5%) and linalyl acetate in *L. hybrida* (26.8%). The EO of *I. verum* was characterized by the presence of a good amount of anethol (89.8%). More represented constituents of *L. cubeba* were neral and geranial (32.5% and 36.4%, respectively), while *R. officinalis* was characterized by 37.9% of *α*-pinene, followed by 1,8-cineole (22.0%).

### 3.2. Antibacterial Activity

The results obtained in this investigation showed that the assayed EOs had different degrees of growth inhibition against the three tested bacterial isolates (Table 2). 

*A. nobilis*, *I. verum*, *L. hybrida*, and *L. cubeba* had no activity against the three tested bacteria.

*S. sclarea* showed good activity against *S. pseudointermedius*, with an MIC value of 2.23 µg/µL and scarce activity against *P. aeruginosa* (MIC of 17.86 µg/µL). *O. vulgare* inhibited the growth of *S. aureus* and *S. pseudointermedius*, with MIC values of 2.36 µg/µL and 1.18 µg/µL, respectively; on the other hand, no activity was observed against *P. aeruginosa*. Very low antimicrobial activity was observed for the remaining EOs. DMSO, tested as negative control, did not cause growth inhibition.

*P. aeruginosa* was resistant to eleven antibiotics and susceptible only to amikacin; *S. aureus* were susceptible to tetracycline, amoxicillin–clavulanic acid, erythromycin, and sulphametoxazole–trimethoprim; *S. pseudointermedius* was susceptible only to amoxicillin–clavulanic acid (Table 3).

### 3.3. Antimycotic Activity

Tested fungal isolates showed different sensitivity patterns to assayed EOs. The overall lowest MIC values were reported for *O. vulgare* and *R. officinalis*. *S. sclarea, R. officinalis*, and *T. vulgaris* had MIC values <0.3 µg/µL against *C. tropicalis*, while *O. vulgare* showed a good antifungal activity against aspergilli. *Trichosporon* sp. appeared to be sensitive to *R. officinalis* only, while *Rhodotorula* sp. was inhibited from *R. officinalis* and *O. vulgare*. This latter EO was the sole active when tested against *C. albicans.* Detailed results are reported in Table 4.

## 4. Discussion

The bacterial strains analyzed in this study have previously been isolated from pets with chronic otitis externa. These strains resulted multiresistant to several antibiotics, and for this reason they caused problems in therapy. 

*S. aureus* has been isolated from several body sites of dogs and cats—in particular from skin, anterior nares, and anal region. Staphylococci seem to be transferred from the nose and mouth of the dog to its coat and ears by grooming and pruritic behaviors [20]. 

*S. pseudointermedius* is part of the microflora of the upper respiratory tract, mucosal, and skin surfaces of dogs and cats; moreover, it may be an invasive pathogen determining some pathologies, including otitis. Resistance of *S. aureus* and *S. pseudintermedius* to classical antibiotics used in clinical veterinary therapy is an increasing threat [21].

*P. aeruginosa* is an opportunistic, ubiquitous bacterial pathogen that can be isolated from the tissue of healthy animals, and has been determined to be the distinct cause of several infections, including chronic otitis externa. An increasing number of *P. aeruginosa* strains resistant to several antibiotics has been observed in recent years [22].

The results of this study show that not all EOs have activity against the tested bacterial strains.

EO of *O. vulgare* gave good results only against staphylococci, in accordance with other studies that consider this EO as an effective antistaphylococcal natural product.

The major compounds of oregano EO—carvacrol, thymol, and terpenoids—seem to determine the antimicrobial activity [23], and it is speculated that they disrupt the membrane of bacteria; in particular, they sensitize the phospholipid bilayer of the cell membrane, increasing the permeability and leakage of vital intracellular constituents [24].

Previous studies found that *S. sclarea* is active against staphylococci, including *S. aureus* [25], whereas we found that the tested *S. aureus* strain was resistant. However, data about the antimicrobial activity of *S. sclarea* are mainly related to food pathogens, whereas no information is available about its clinical use.

*Trichosporon* sp., *C. albicans*, and *A. terreus* were insensitive to most EOs evaluated in the present study, while other fungi showed different degrees of sensitivity. *C. tropicalis* was reported as sensitive when tested against *S. sclarea, R. officinalis*, and *T. vulgaris*, confirming previously reported data concerning this latter EO [15,26]. 

*O. vulgare* has a known antimicrobial effect [26,27] due to the high amount of carvacrol, confirming the results of our study. 

*R. officinalis* yielded interesting results showing its efficacy, which appear to be in agreement with Fu et al. [28] for *A. niger*, but not for *C. albicans.* Our results confirm the effectiveness of *O. vulgare* against *A. niger* and *Rhodotorula* sp., and the ineffectiveness of *I. verum* against *A. niger*, reported by Elgayyar et al. [27], but would not corroborate data referred to both *O. basilicum* and *R. officinalis,* as well as the efficacy of *I. verum* against *Rhodotorula* sp. 

Molds and yeasts tested in the present report were cultured from affected animals’ ears. Even if antifungal drug resistance is not a common feature involving these agents, such alternative treatment based on natural products could achieve the owners’ approval [10], but the choice of an EOs-based treatment should take into account the identification of fungal organisms responsible for the disease in order to select the most suitable product for treatment. In fact, the results obtained in the present study would suggest that the analyzed EOs are not always able to replace antibiotics or antimycotic drugs to treat such infections, being that their effectiveness is strictly related to the pathogen involved. For example, in the case of *P. aeruginosa* isolate, the treatment with amikacin resulted better than the use of EOs that, although active, had high MIC values (from 17.86 µg/µL to 18.34 µg/µL).

In the light of the above considerations, the first choice option would take into account the causative agents of otitis with isolation and typing of the etiologic agent; successively, it would be important to carry out an antimicrobial susceptibility test to select an appropriate therapy. EOs can represent a good therapeutic tool, but considered that their efficacy is strongly related to the bacterial strain, it could be useful to evaluate the in vitro activity of different EOs in order to select a natural product effective against the pathogen and that could improve the efficacy treatment. 

In conclusion, EOs could be scheduled as an alternative therapeutic option in bacterial otitis complicated by fungi in association with conventional drugs.

## Figures and Tables

**Table 1:** Relative percentage of the main constituents of tested essential oils (EOs).

N.	Class	Component	RI	*A.n*	*O.v*	*O.b*	*S.s*	*T.v*	*I.v*	*R.o*	*L.c*	*L.h*
1	est	propyl butanoate	898	5.5								
2	mh	tricyclene	926						1.4		1.5	
3	mh	*α*-pinene	940	1.2						37.9		
4	mh	camphene	953							5.4		
5	mh	sabinene	976								1.0	
6	est	isopropyl tiglate	976	1.7								
7	mh	*β*-pinene	980							5.0	1.2	
8	nt	6-methyl-5-hepten-2-one	985								1.5	
9	mh	myrcene	991		2.2					1.6		
10	est	isobutyl isovalerate	1005	1.3								
11	est	2-methylbutyl isobutyrate	1015	4.5								
12	mh	*α*-terpinene	1018		2.1							
13	mh	o-cymene	1026							1.1		
14	mh	p-cymene	1026		9.3			15.3				
15	mh	limonene	1031						3.9	3.3	16.3	
16	om	1,8-cineole	1033			5.9				22.0	2.3	7.7
17	mh	(Z) *β*-ocimene	1040									1.2
18	mh	(E) *β*-ocimene	1050									2.1
19	est	isobutyl angelate	1053	34.5								
20	mh	*γ*-terpinene	1062		5.3			2.9		0.5		
21	mh	artemisia ketone	1065	7.4								
22	om	cis-linalool oxide (furanoid)	1074				2.2					
23	om	trans-linalool oxide (furanoid)	1088				1.8					
24	est	isobutyl tiglate	1093	1.5								
25	om	trans-sabinene hydrate	1097		1.8			3.8				
26	om	linalool	1098			46.0	8.1				1.5	31.5
27	om	trans-pinocarveol	1142	1.7								
28	om	camphor	1143							7.6		7.3
29	est	propyl tiglate	1153	5.3								
30	est	isoamyl angelate	1162	18.7								
31	om	borneol	1165					1.6		2.0		2.1
32	om	pinocarvone	1166	2.7								
33	om	terpinen-4-ol	1177					2.4				4.0
34	om	α-terpineol	1189									2.1
35		unknown						1.7				
36	pp	methyl chavicol (=estragol)	1195			1.1						
37	om	thymol methyl ether	1232					1.7				
38	om	neral	1240								32.5	
39	om	linalyl acetate	1257				54.7					26.8
40	om	geranial	1270								36.4	
41	pp	(E)-anethol	1283						89.8			
42	om	isobornyl acetate	1285			1.6				3.3		
43	om	bornyl acetate	1285									2.4
44	om	thymol	1290					52.6				
45	om	carvacrol	1298		65.9							
46	om	(E)-8-hydroxylinalool	1345				5.6					
47	om	(Z)-8-hydroxylinalool	1360				15.8					
48	pp	eugenol	1356			11.5						
49	sh	*β*-elemene	1392			2.2						
50	sh	*β*-caryophyllene	1418		3.7			6.8		4.1		2.2
51	sh	trans-α-bergamotene	1437			3.6						
52	sh	*α*-guaiene	1440			1.1						
53	sh	(E)-*β*-farnesene	1458									1.4
54	sh	germacrene D	1481			3.5						
55	sh	*α*-bulnesene	1505			2.0						
56	sh	trans-*γ*-cadinene	1513			2.8						
57	sh	*δ*-cadinene	1524					1.0				
58	os	caryophyllene oxide	1581				4.8			0.3		
59	os	1,10-di-epi-cubenol	1614			1.0						
60	os	t-cadinol	1640			5.8						
61	od	sclareol	2223				1.3					
Total%				92.1	98.5	99.2	98.9	95.6	100.0	97.4	99.4	100.0

Legend: RI: Retention index measured on HP-5 column; Compounds present in percentage <1% were excluded from the table. *A.n*.: *Anthemis nobilis*; *I.v*.: *Illicium verum*; *L.h*.: *Lavandula hybrid*; *L.c*.: *Litsea cubeba*; *O.b*.: *Ocimum basilicum*; *O.v*.: *Origanum vulgare* subsp. *hirticum; R.o*.: *Rosmarinus officinalis*; *S.s*.: *Salvia sclarea*; *T.v*.: *Thymus vulgaris*; mh: monoterpene hydrocarbons; om: oxygenated monoterpenes; sh: sesquiterpene hydrocarbons; os: oxygenated sesquiterpenes; nt: non-terpenes; pp: phenylpropanoids; od: oxygenated diterpenes; est: non-terpenic esters.

**Table 2:** The inhibition zones obtained testing the selected bacterial strains against the assayed EOs.

		Bacterial Strains	
	*Staphylococcus aureus*	*Staphylococcus pseudointermedius*	*Pseudomonas aeruginosa*
**Essential Oils**			
	M SD	M SD	M SD
*Anthemis nobilis*	0.0 ± 0.0	0.0 ± 0.0	0.0 ± 0.0
*Illicium verum*	0.0 ± 0.0	0.0 ± 0.0	0.0 ± 0.0
*Lavandula hybrida*	0.0 ± 0.0	0.0 ± 0.0	0.0 ± 0.0
*Litsea cubeba*	0.0 ± 0.0	0.0 ± 0.0	0.0 ± 0.0
*Ocimum basilicum*	0.0 ± 0.0	7.0 ± 0.0 ^1^	8.0 ± 0.0 ^2^
*Origanum vulgare*	13.3 ± 0.6 ^3^	17.3 ± 0.6 ^4^	0.0 ± 0.0
*Rosmarinus officinalis*	0.0 ± 0.0	0.0 ± 0.0	7.0 ± 0.0 ^5^
*Salvia sclarea*	0.0 ± 0.0	14.3 ± 0.6 ^6^	8.0 ± 0.0 ^7^
*Thymus vulgaris*	8.3 ± 0.6 ^8^	0.0 ± 0.0	0.0 ± 0.0

Legend: M: mean expressed in mm; MIC: minimum inhibitory concentration; SD: standard deviation. ^1^ MIC: 18.34 µg/µL; ^2^ MIC: 18.34 µg/µL; ^3^ MIC: 2.36 µg/µL; ^4^ MIC: 1.18 µg/µL; ^5^ MIC: 18.28 µg/µL; ^6^ MIC: 2.23 µg/µL; ^7^ MIC: 17.86 µg/µL; ^8^ MIC: 18.73 µg/µL.

**Table 3:** The inhibition zones (expressed in mm) obtained testing the selected bacterial strains against different antibiotics (standard deviation was equal to 0).

		Bacterial Strains	
	*Staphylococcus aureus*	*Staphylococcus pseudointermedius*	*Pseudomonas aeruginosa*
**Antibiotics**			
**Amikacin**	7 (R)	9 (R)	17 (S)
**Amoxycillin–clavulanic acid**	27 (S)	26 (S)	0 (R)
**Ampicillin**	16 (R)	15 (R)	0 (R)
**Ceftazidime**	0 (R)	0 (R)	0 (R)
**Cephalexin**	0 (R)	0 (R)	0 (R)
**Cephotaxime**	0 (R)	0 (R)	13 (R)
**Ciprofloxacin**	10 (R)	0 (R)	0 (R)
**Enrofloxacin**	14 (R)	0 (R)	0 (R)
**Erythromycin**	17 (S)	0 (R)	0 (R)
**Streptomycin**	0 (R)	0 (R)	0 (R)
**Sulphametoxazole–trimethoprim**	24 (S)	0 (R)	0 (R)
**Tetracycline**	20 (S)	0 (R)	0 (R)

Legend: S: susceptible; R: resistant.

**Table 4:** MIC values (expressed in µg/µL) of tested EOs versus selected fungal agents (standard deviation was equal to 0).

Fungal Agents							
Essential Oils	*Candida albicans*	*Candida tropicalis*	*Aspergillus niger*	*Aspergillus terreus*	*Aspergillus fumigatus*	*Trichosporon* sp.	*Rhodotorula* sp.
*Anthemis nobilis*	4.50	4.50	4.50	4.50	2.25	4.50	4.50
*Illicium verum*	2.44	4.88	2.44	2.44	0.59	9.78	2.44
*Lavandula hybrida*	4.25	4.25	2.12	>8.50	8.50	>8.50	4.25
*Litsea cubeba*	4.43	2.21	0.53	2.21	0.18	8.86	4.43
*Ocimum basilicum*	2.29	4.58	1.10	4.58	2.29	9.17	1.10
*Origanum vulgare*	0.19	1.14	0.19	0.19	0.19	2.37	0.19
*Rosmarinus officinalis*	9.14	0.29	0.29	>9.14	0.29	0.46	0.29
*Salvia sclarea*	1.07	0.18	2.23	4.46	2.23	>8.93	>8.93
*Thymus vulgaris*	5.72	0.19	0.56	11.71	5.72	11.71	5.72

## Supplementary Materials

Supplementary File 1
