# Supplementary figures and images for: Antibacterial and Antifungal Activity of Essential Oils against Pathogens Responsible for Otitis Externa in Dogs and Cats
Source: Medicines (Basel). 2017 Apr 21;4(2):21. doi: 10.3390/medicines4020021 (PMC5590057; doi:10.3390/medicines4020021)

*BACTERIA*

*FUNGI*

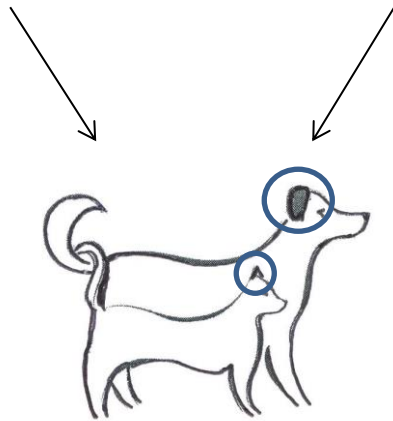

OTITIS EXTERNA

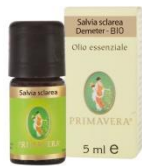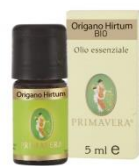

ESSENTIAL  
OILS

Supplement: Supplementary File 1 [file medicines-04-00021-s001.pdf]
